# Supplementary material for: Superfast precipitation of energetic electrons in the radiation belts of the Earth
Source: Nat Commun. 2022 Mar 25;13:1611. doi: 10.1038/s41467-022-29291-8 (PMC8956639; doi:10.1038/s41467-022-29291-8)
Supplement: Supplementary file 1 — Supplementary Information [file 41467_2022_29291_MOESM1_ESM.pdf]

Supplementary Information for "Superfast Precipitation of Energetic Electrons in the Radiation Belts of the Earth"

Xiao-Jia Zhang[1*], Anton Artemyev[1], Vassilis Angelopoulos[1], Ethan Tsai[1], Colin Wilkins[1], Satoshi Kasahara[2], Didier Mourenas[3], Shoichiro Yokota[4], Kunihiro Keika[2], Tomoaki Hori[5], Yoshizumi Miyoshi[5], Iku Shinohara[6], and Ayako Matsuoka[7]

[1]Department of Earth, Planetary, and Space Sciences, University of California, Los Angeles, California, 90095, USA
[2]Department of Earth and Planetary Science, School of Science, The University of Tokyo, Tokyo, Japan
[3]Laboratoire Matière en Conditions Extrêmes, Paris-Saclay University, CEA, Bruyères-le-Châtel, France
[4]Department of Earth and Space Science, Graduate School of Science, Osaka University, Toyonaka, Japan
[5]Institute for Space Earth Environmental Research, Nagoya University, Nagoya, Japan
[6]Institute of Space and Astronautical Science, Japan Aerospace Exploration Agency, Sagamihara, Japan
[7]Graduate School of Science, Kyoto University, Kyoto, Japan

[*] Corresponding Author: Xiao-Jia Zhang, xjzhang@ucla.edu

March 2, 2022

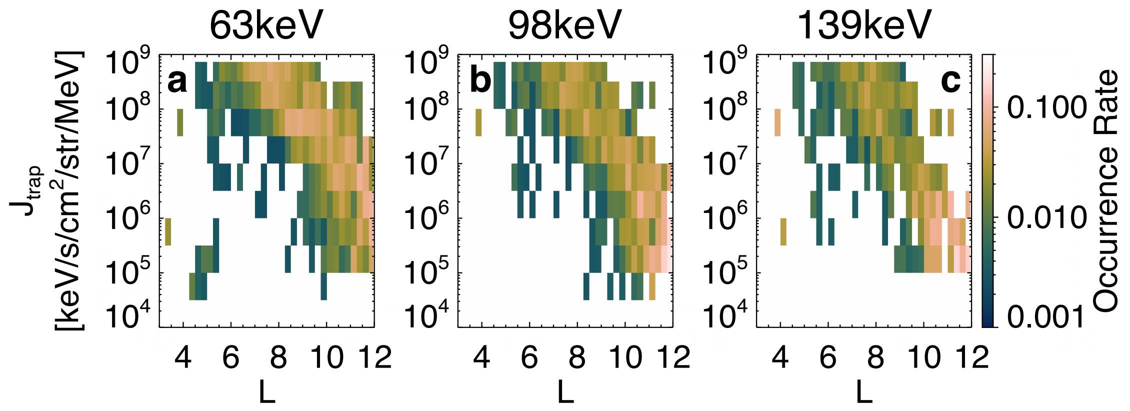

Supplementary Fig. 1: **Occurrence rate of loss-cone overfilling, $j_{prec}/j_{trap} > 1$, from ELFIN measurements from September 2020 to January 2021.** Results of three energy channels are shown in the plane of L-shell (equatorial radial distance from Earth in Earth radii) and trapped flux magnitude.

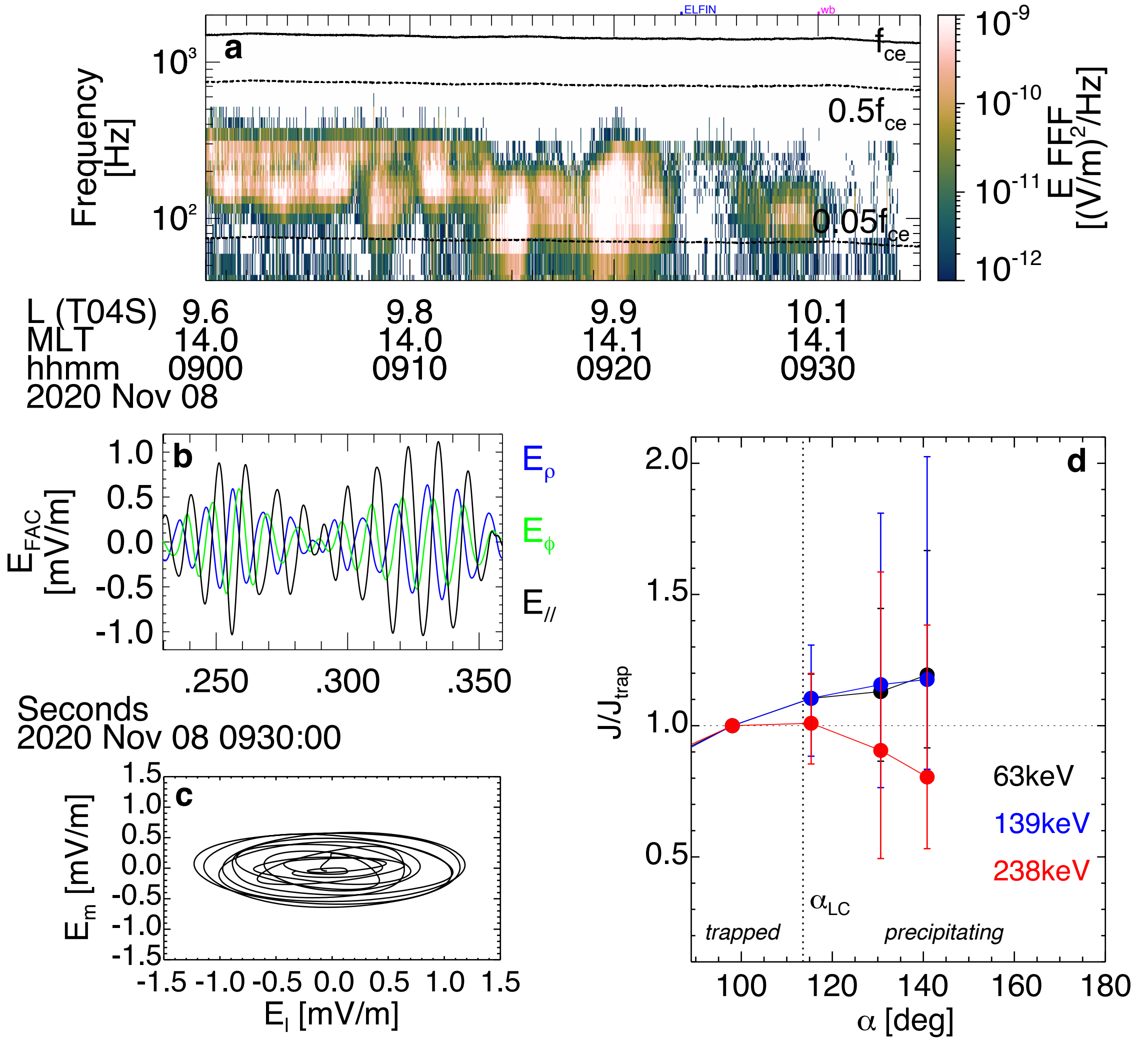

Supplementary Fig. 2: **One example of loss-cone overfilling observations by ELFIN-A on 08 November 2020, in conjunction with equatorial wave measurements by THEMIS-E.** (**a**) Electric field spectrum in the whistler-mode frequency range with indications of the times of closest conjunction with ELFIN (blue bar) and with wave-burst (wb) measurements (magenta bar), (**b**) examples of wave packets in the field-aligned coordinate system during the wave-burst intervals, (**c**) electric field hodogram of intermediate variance component (E_{m}) versus maximum component (E_{l}) for the wave packets in panel (b), (**d**) pitch-angle (α) distributions of 63 keV (black), 139 keV (blue), and 238 keV (red) electron fluxes normalized to 90° fluxes (averaged over intervals with loss-cone overfilling observed at ELFIN around the times of conjunction with THEMIS), j/j_{trap}. In panel (d), the vertical dotted line indicates the local loss cone angle, α_{LC}, which separates the trapped and precipitating electrons; the horizontal dotted line denotes $j/j_{\mathrm{trap}} = 1$; the error bars mark the minimum and maximum j/j_{trap} for precipitating electrons during the loss-cone overfilling interval.

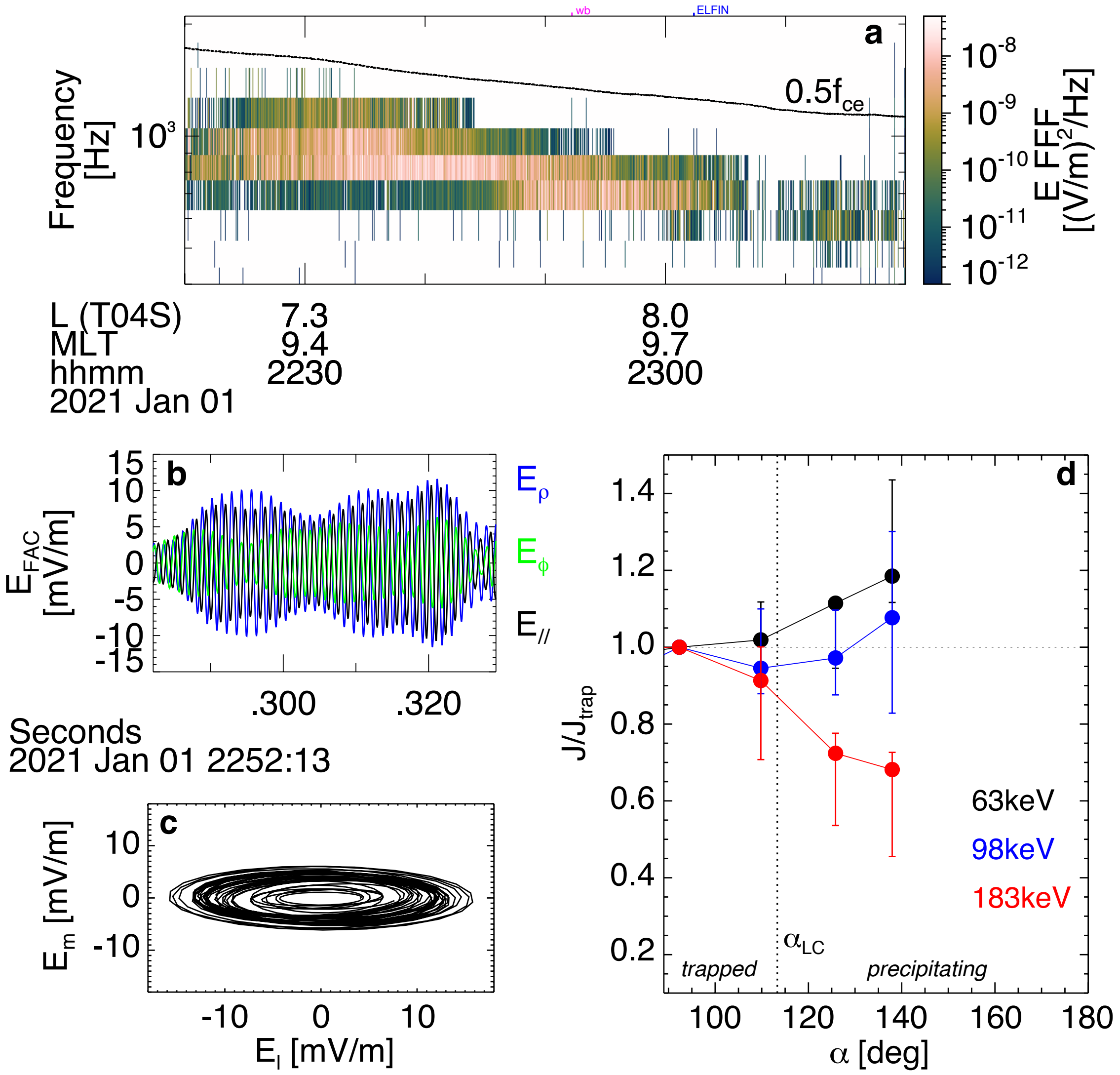

Supplementary Fig. 3: One example of loss-cone overfilling observations by ELFIN-A on 01 January 2021, in conjunction with equatorial wave measurements by THEMIS-E. (**a**) Electric field spectrum in the whistler-mode frequency range with indications of the times of closest conjunction with ELFIN (blue bar) and with wave-burst (wb) measurements (magenta bar), (**b**) examples of wave packets in the field-aligned coordinate system during the wave-burst intervals, (**c**) electric field hodogram of intermediate variance component ($E_{\rm m}$) versus maximum component ($E_{\rm l}$) for the wave packets in panel (b), (**d**) pitch-angle (α) distributions of 63 keV (black), 98 keV (blue), and 183 keV (red) electron fluxes normalized to 90° fluxes (averaged over intervals with loss-cone overfilling observed at ELFIN around the times of conjunction with THEMIS), $j/j_{\rm trap}$. In panel (d), the vertical dotted line indicates the local loss cone angle, $\alpha_{\rm LC}$, which separates the trapped and precipitating electrons; the horizontal dotted line denotes $j/j_{\rm trap} = 1$; the error bars mark the minimum and maximum $j/j_{\rm trap}$ for precipitating electrons during the loss-cone overfilling interval.

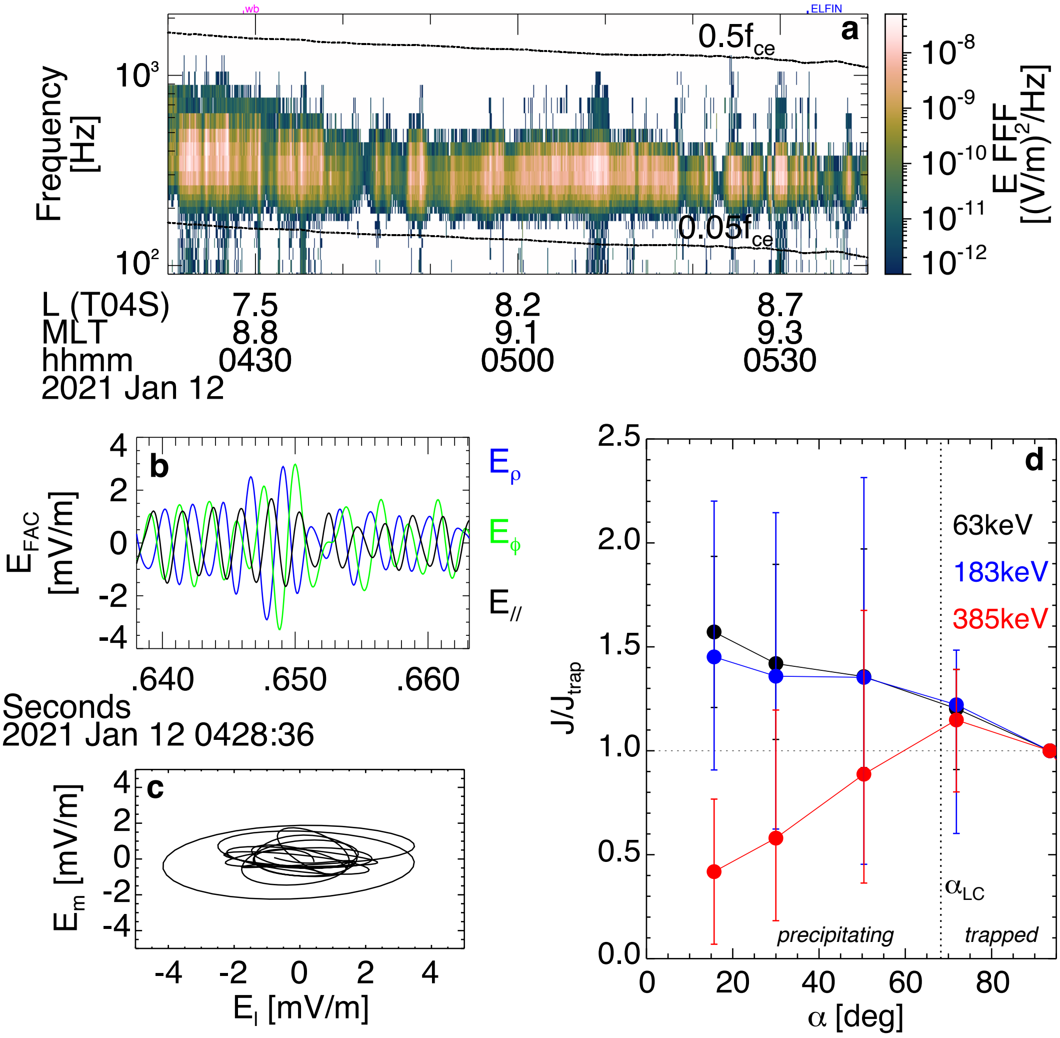

Supplementary Fig. 4: **One example of loss-cone overfilling observations by ELFIN-A on 12 January 2021, in conjunction with equatorial wave measurements by THEMIS-E.** (**a**) Electric field spectrum in the whistler-mode frequency range with indications of the times of closest conjunction with ELFIN (blue bar) and with wave-burst (wb) measurements (magenta bar), (**b**) examples of wave packets in the field-aligned coordinate system during the wave-burst intervals, (**c**) electric field hodogram of intermediate variance component (E_{m}) versus maximum component (E_{l}) for the wave packets in panel (b), (**d**) pitch-angle (α) distributions of 63 keV (black), 183 keV (blue), and 385 keV (red) electron fluxes normalized to 90° fluxes (averaged over intervals with loss-cone overfilling observed at ELFIN around the times of conjunction with THEMIS), j/j_{trap}. In panel (d), the vertical dotted line indicates the local loss cone angle, α_{LC}, which separates the trapped and precipitating electrons; the horizontal dotted line denotes $j/j_{\mathrm{trap}} = 1$; the error bars mark the minimum and maximum j/j_{trap} for precipitating electrons during the loss-cone overfilling interval.

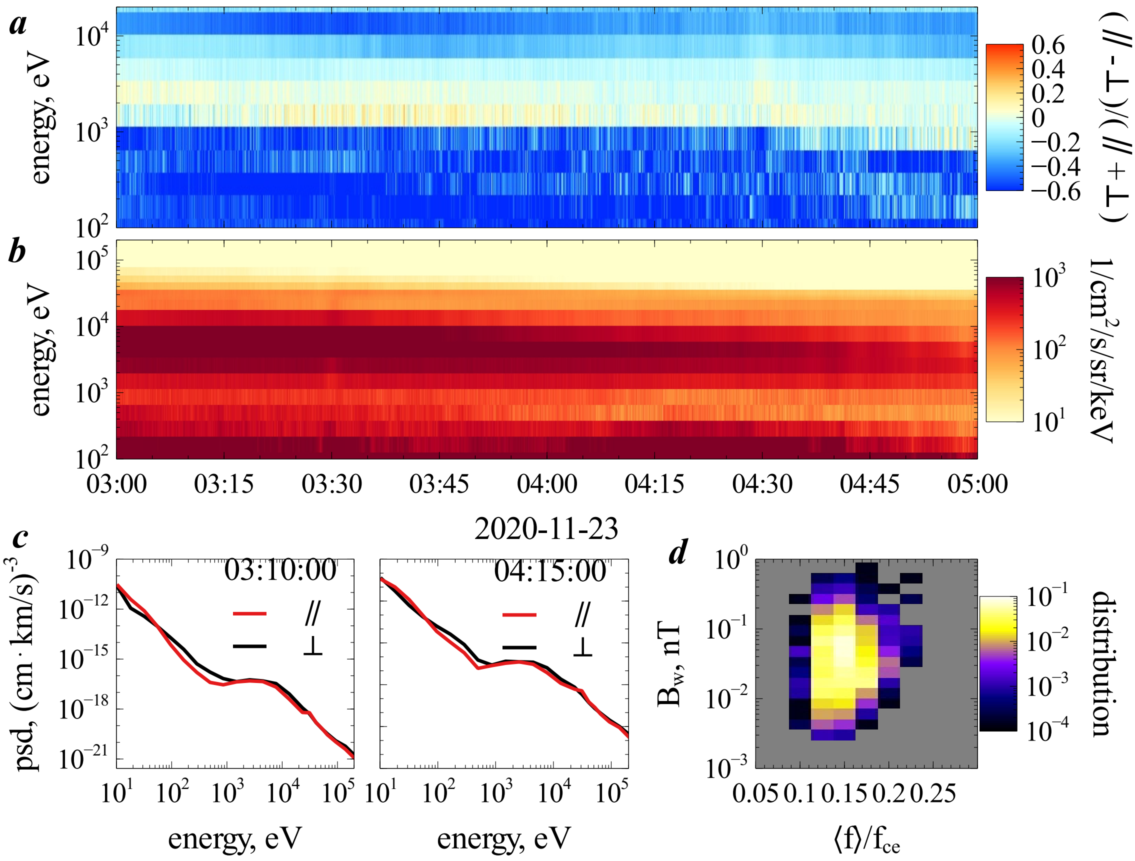

Supplementary Fig. 5: **Overview of THEMIS-E observations on 23 November 2020. a**, Ratio of field-aligned ($\|$ with pitch angles $< 30°$) and transverse ($\perp$ with pitch angles $\in [75, 105]°$) spectra for < 25 keV electrons. **b**, Energy spectra of electron omnidirectional fluxes for < 500 keV. **c**, Examples of electron phase space density (psd) distributions in field-aligned ($\|$) and transverse ($\perp$) directions. **d**, Whistler-mode wave parameter distribution in the plane of wave amplitude $B_w = \sqrt{\langle B_w^2 \rangle}$ versus frequency $\langle f \rangle$ calculated from *fff* spectra. Wave amplitude is rescaled based on the waveform data to represent actual wave packet amplitudes.

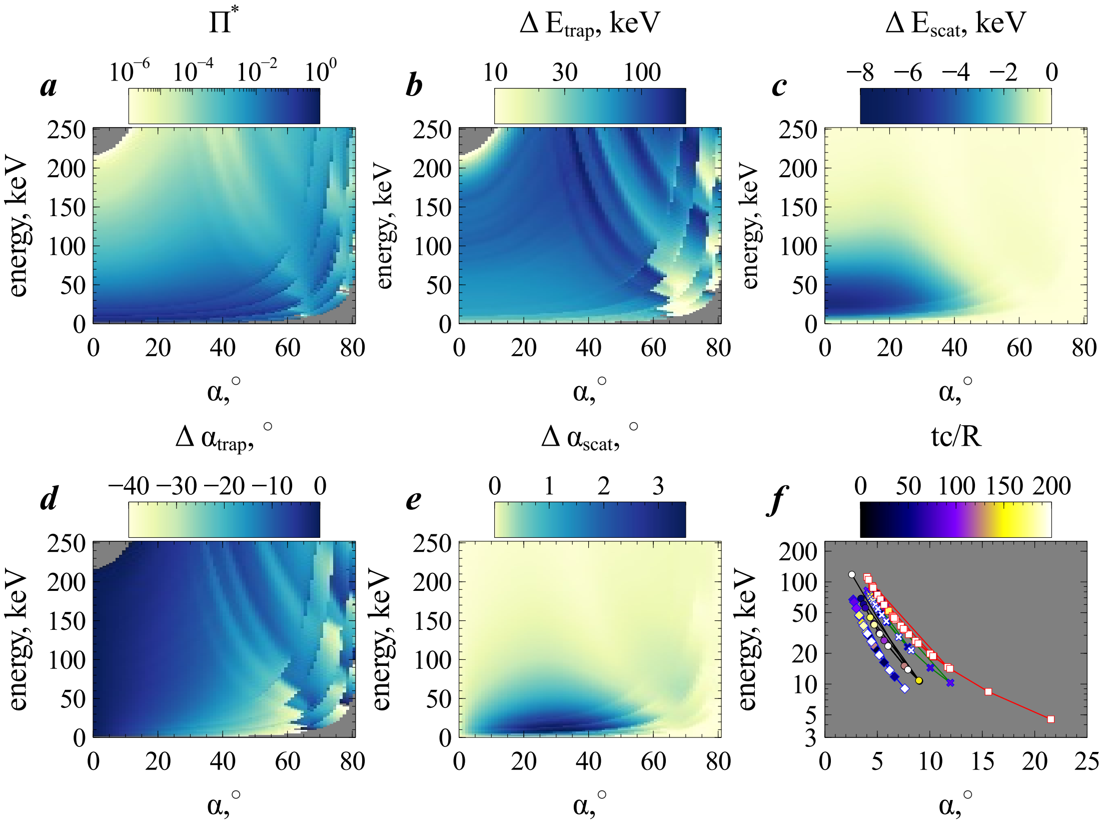

Supplementary Fig. 6: **Overview of results from numerical simulations.** Integral maps (in the plane of electron energy versus pitch angle, α) of (**a**) trapping probability, (**b**) trapping energy change, (**c**) scattering energy change, (**d**) trapping pitch-angle change, (**e**) scattering pitch-angle change, and (**f**) three examples of electron trajectories calculated with the mapping technique (each resonant interaction is marked by a symbol).

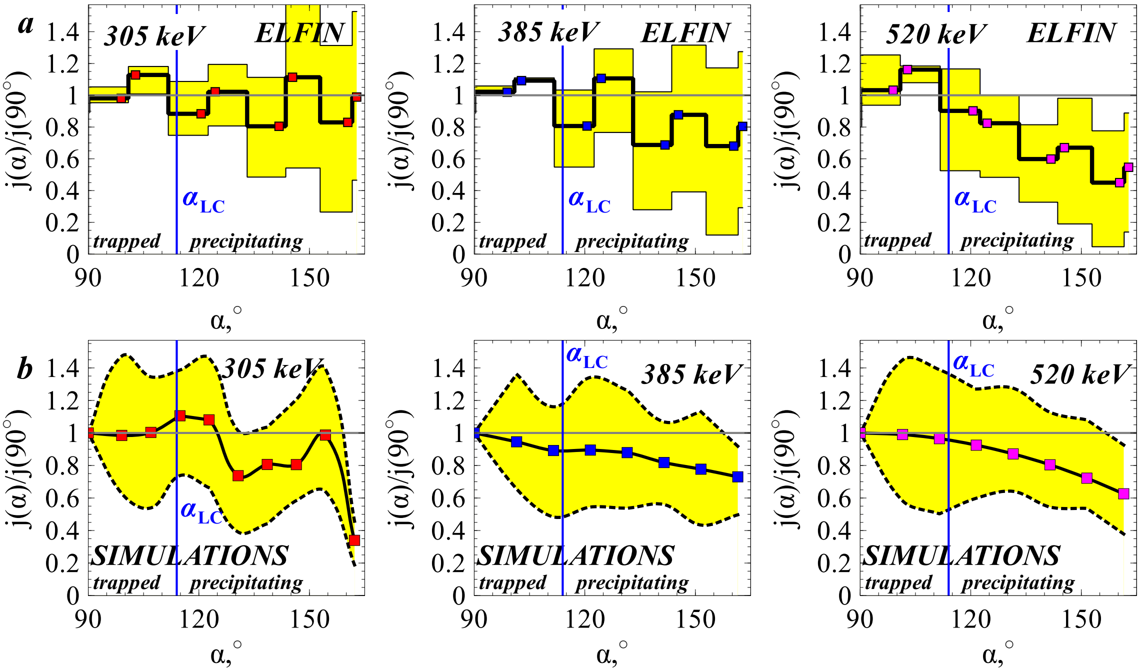

Supplementary Fig. 7: **Comparison of precipitating electron fluxes (in the loss cone) from observations and from numerical simulations, a**, Local pitch-angle (α) spectra of 305 keV, 385 keV, and 520 keV electron fluxes measured by ELFIN spacecraft, averaged over the most intense precipitation interval (from 03:02:01 to 03:02:14 UT), then normalized to the 90° flux average, $j(\alpha)/j(90^\circ)$. **b**, $j(\alpha)/j(90^\circ)$ from numerical simulations of nonlinear electron Landau resonant interaction with oblique whistler-mode waves. Yellow regions indicate the highest and lowest flux ratios during the averaging interval. Blue vertical lines in the pitch angle spectra indicate the local loss cone angle, α_{LC}, which separates the trapped and precipitating electrons. Simulations are performed with probability distributions shown in Supplementary Fig. 6, but for an energy range up to 1 MeV.
